# Multilayer and crushed stent visualization using photon- counting detector computed tomography – a proof of concept

**DOI:** 10.1038/s41598-025-25119-9

**Published:** 2025-10-27

**Authors:** Eva Harmel, Simon Hellbrueck, Dario Bongiovanni, Philip Raake, Thomas Kroencke, Josua A. Decker, Daniel O. Bittner

**Affiliations:** 1 Department of Internal Medicine I, Cardiology, University Hospital Augsburg, Augsburg, Germany; 2 Department of Diagnostic and Interventional Radiology, University Hospital Augsburg, Augsburg, Germany; 3 Department of Cardiology, Friedrich- Alexander University Erlangen- Nürnberg (FAU), Erlangen, Germany

**Keywords:** Photon counting detector computed tomography, Ultra high resolution, Bifurcation stent visualization, Cardiology, Diseases, Engineering, Medical research

## Abstract

Photon‑counting detector CT (PCD‑CT) enables ultra‑high resolution (UHR, 0.2 mm) and may improve visualization of coronary stents compared with standard‑resolution (SR, 0.4 mm). In this ex- vivo study, we utilized a phantom heart model to simulate physiological attenuation and imitated the most common coronary bifurcation stenting techniques by creating multiple stent layers and stent- crush situations (single layer, crushed, two‑ and three‑layer). We compared UHR and SR PCD‑CT using Bv72 and Bv56 kernels. Objective endpoints were in‑stent lumen visibility, signal-to-noise ratio, and percentage change in stent attenuation; subjective image quality was rated on a 5‑point Likert scale (1 = excellent to 5 = non‑diagnostic). Depending on distribution, two‑sided t‑tests (mean ± SD) or Mann–Whitney U tests (median[IQR]) were applied, p‑values were Bonferroni‑corrected for multiple comparisons. In the pooled Crush cohort, UHR Bv72 improved in‑stent lumen visibility when compared with both, SR Bv72 (single-layer: 68.08 ± 4.30% vs. 79.03 ± 3.79%; crushed: 63.62 ± 4.97% vs. 74.73 ± 2.51%) and SR Bv56 (single-layer: 63.63 ± 4.47% vs. 79.03 ± 3.79%; crushed: 62.18 ± 3.55% vs. 74.73 ± 2.51%), all *p* < 0.01. The effect was even more pronounced in small stents (single‑layer 65.45 ± 1.91% (SR) vs. 77.11 ± 2.55% (UHR) and crushed 60.67 ± 2.67% vs. 74.00 ± 2.67%), both *p* < 0.01. Regarding subjective image quality, UHR was associated with better sharpness and less blooming, e.g., UHR Bv72 vs. SR Bv56 (multilayer: sharpness 1[0] vs. 2[0], blooming 1[0] vs. 2[0]; crush: sharpness 1[0] vs. 2[0], blooming 1[0] vs. 2[1]; all *p* ≤ 0.01) and UHR Bv72 vs. SR Bv72 (multilayer: sharpness 1[0] vs. 1[1], blooming 1[0] vs. 1[1]; crush: sharpness 1[0] vs. 1[1]; all *p* ≤ 0.05). These findings indicate that UHR PCD‑CT with Bv72 might enable assessment of complex stents in a phantom setting; even though clinical studies are warranted.

**Supplementary Information:**

The online version contains supplementary material available at 10.1038/s41598-025-25119-9.

## Introduction

Coronary artery disease (CAD) remains a leading cause of morbidity and mortality worldwide, affecting over 18 million adults in the United States^1,2^. Percutaneous coronary intervention (PCI) with stent implantation has revolutionized the treatment of obstructive coronary artery disease^3^. Nonetheless, de- novo stenosis but also in- stent restenosis and stent thrombosis still affect event- free survival, necessitating careful follow- up and reevaluation^4^. Invasive coronary angiography has long been considered the gold standard for assessing stent patency, but it carries risks associated with its invasive nature^5^. Non- invasive imaging modalities, particularly coronary computed tomography angiography (CCTA), have emerged as potential alternative, with a class I recommendation for first line imaging to evaluate chronic coronary syndrome in patients with low to intermediate risk for CAD^6^. However, energy-integrating detector computed tomography (EID-CT) remains limited in its ability to visualize high calcium burden or coronary stents, owing to physical artifacts such as beam hardening and to image degradation from blooming artifacts and partial volume effects related to limited spatial resolution^7,8^. Historically, visualizing metallic structures, such as coronary stents within coronary vessels, posed significant challenges with EID- CT, often leading to patient exclusion from non- invasive CCTA in favor of invasive coronary angiography^7,8^. This issue was particularly pronounced in complex cases involving coronary bifurcation stents with several stent strut layers, leading to more blooming artifacts, and reduced in- stent lumen visibility^9^. With the advent of photon counting detector computed tomography (PCT- CT) systems in 2021, several limitations inherent to traditional EID- CT systems have been addressed^10,11^. Additionally, the novel implementation of ultra- high resolution (UHR) mode of PCD- CT further pushes the boundaries of spatial resolution, potentially allowing for more accurate stent lumen assessment^12^.

Therefore, we sought to evaluate multilayer and crushed stent situations – as seen in bifurcation stent techniques – comparing novel UHR and standard resolution (SR) scan mode PCD- CT. As a proof of concept, in an ex vivo phantom model, we hypothesize that UHR scans will provide better image quality and in- stent lumen visibility due to its improved spatial resolution and reduced artifacts.

## Methods

This study was conducted using only phantom data; no human or animal subjects were involved. Therefore, institutional ethics approval was not required.

### Phantom setup

For this study, we utilized an anthropomorphic thoracic phantom (Model 008 C, Computerized Imaging Reference Systems Inc., Virginia, USA) designed with tissue- equivalent materials to replicate physiological X- ray attenuation^13^. The phantom features a removable cylindrical component that houses the heart, which can be opened to access predefined cutouts simulating the left coronary artery. Within these cutouts, we individually positioned inserts to model various stent scenarios, as detailed in the subsequent section.

All stents were deployed inside rigid plastic tubes with a 5 mm diameter using standard balloon- expansion techniques. To achieve physiological attenuation, the tubes were filled with a diluted contrast medium (Iopromide, Ultravist 300 mg/ml, Bayer Vital, Leverkusen, Germany) until the measured Hounsfield Units (HU) matched reference values obtained from in vivo CCTA. Both tube ends were securely sealed with wax plates and adhesive to prevent leakage.

### Study design

**Fig. 1:**
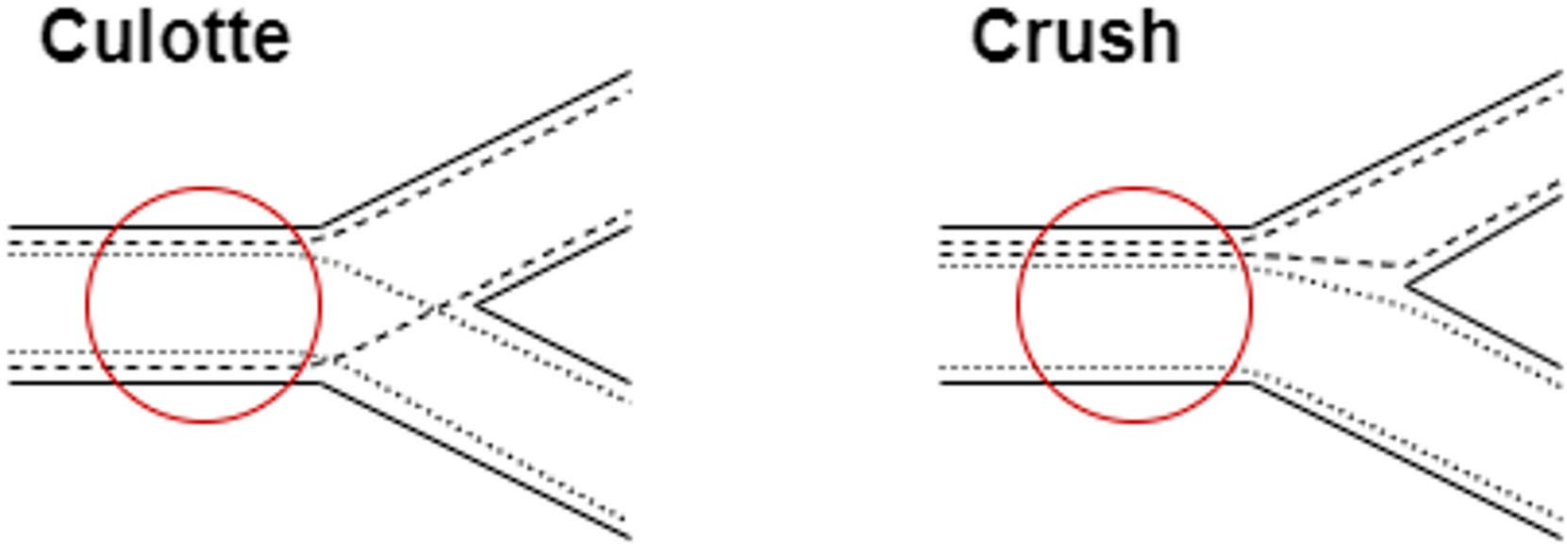
Culotte vs. Crush technique for bifurcation stenting.

**Fig. 2:**
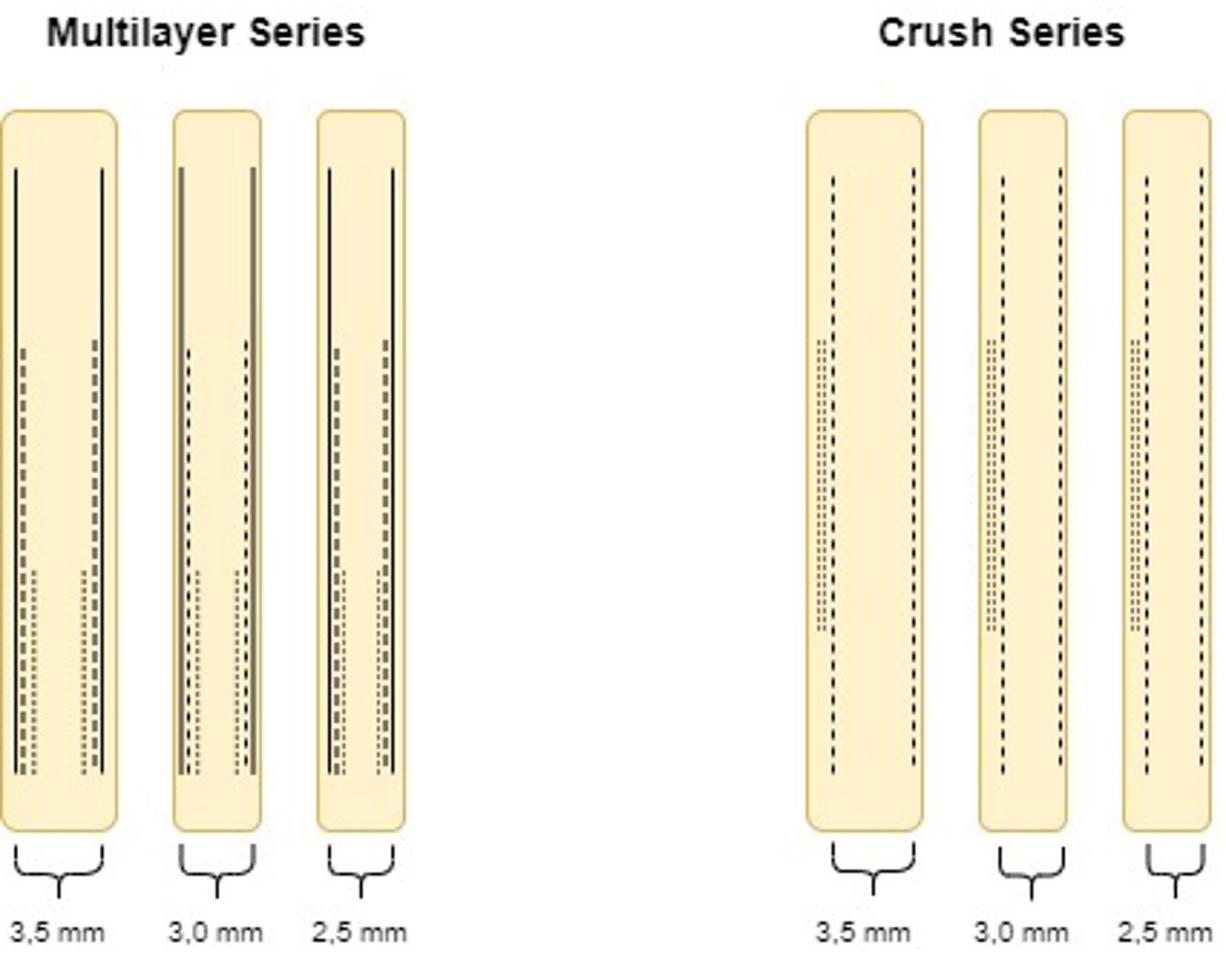
Stent deployment in Crush vs. Multilayer Series.

For our analysis we focused on the two widely used two stent strategies for the treatment of bifurcation stenosis, namely the *Culotte* and the *Crush* technique Figure 1. To model these techniques, we designed two distinct series of stent implantations, each mimicking the symmetrical and asymmetrical configurations of stent layers in the proximal vessel, as seen in the *Culotte* and the *Crush* technique, respectively Figure 2.

For both series, we exclusively used a single stent type – the Promus PREMIER™ Select (Boston Scientific Corporation, Marlborough, MA, USA), an everolimus- eluting, platinum- chromium coronary stent system. This stent has a strut thickness of 0.093 mm for diameters smaller than 4.0 mm.

### Multilayer stent series

In the *Culotte* technique, the first stent is deployed from the main vessel into the side branch, as displayed in Fig. 1. Following this, a second stent is then placed inside the first, extending distally into the main branch. This configuration results in two layers of stent material covering the proximal main vessel Figure 1.

To imitate the symmetrical, multilayer stent configuration characteristic of the *Culotte* technique, we implanted coronary stents of various lengths (i.e., 12 mm, 20 mm and 28 mm) into plastic tubes, selecting tube diameters closely matched to the target stent sizes (3.0 mm tubes for 2.5 mm and 3.0 mm stents, and 4.0 mm tubes for 3.5 mm stents). Each stent was deployed at nominal inflation pressures recommended by the manufacturer’s compliance chart, creating the “*multilayer stent series*” with one, two, and three symmetrical stent layers for each stent size.

### Crush stent series

In contrast, the *Crush* technique involves deploying the first stent from the side branch into the main vessel, with the proximal portion of this stent subsequently crushed against the vessel wall using a balloon. A second stent is then placed from the proximal main vessel into the distal main branch, creating an asymmetrical stent layer configuration in the proximal segment of the main vessel, with three stent layers on one side and a single layer on the other Figure 1.

To imitate this asymmetric configuration, we designed the “*crush stent series*”. In this model, we first implanted a shorter stent (12 mm length) and crushed it against the side of the plastic tube using a non- compliant balloon sized to the tube’s diameter (NC 4.0/15 mm for the 4.0 mm tubes and NC 3.0/15 mm for the 3.0 mm tubes). We then implanted a second, longer stent (28 mm length) overlying the crushed stent in the proximal main vessel. This approach was applied to each stent diameter (i.e., 2.5 mm, 3.0 mm, and 3.5 mm).

### CT scan protocol and image reconstruction

All scans were performed on a first generation, dual- source PCD- CT (NAEOTOM Alpha, Siemens Healthineers, Erlangen, Germany). Specific characteristics of the PCD- CT are previously described by Decker et al.^10^. Each stent configuration was scanned two separate times: (1) a retrospectively ECG- gated spiral CT with a standard resolution (SR) scan mode with a collimation of 144 × 0.4 mm^2^ and (2) a retrospectively ECG- gated spiral CT with the novel ultra- high resolution (UHR) acquisition mode with a collimation of 120 × 0.2 mm^2^ and a simulated ECG of 60 bpm. The tube voltage was set to 120 kVp with a CARE keV IQ level of 64 to adjust the tube current- time product.

**Fig. 3:**
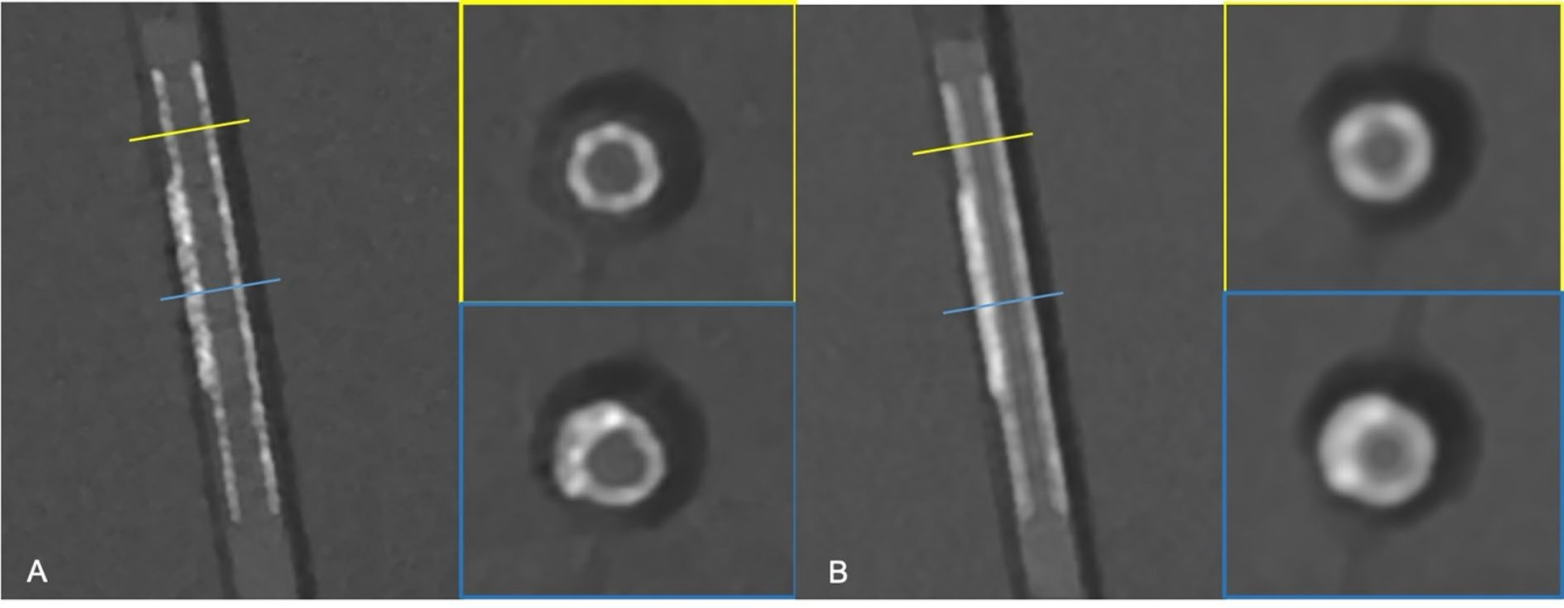
The image shows a 2.5 mm stent of the “Crush Series” in (**A**) Bv72 kernel reconstruction with a 0.2 mm slice thickness (“ultra- high resolution”) and (**B**) Bv56 kernel reconstruction with a 0.4 mm slice thickness. The blue and yellow line correlate with the horizontal images on the right, showing a single- layer stent zone (yellow) vs. crush zone (blue).

**Fig. 4:**
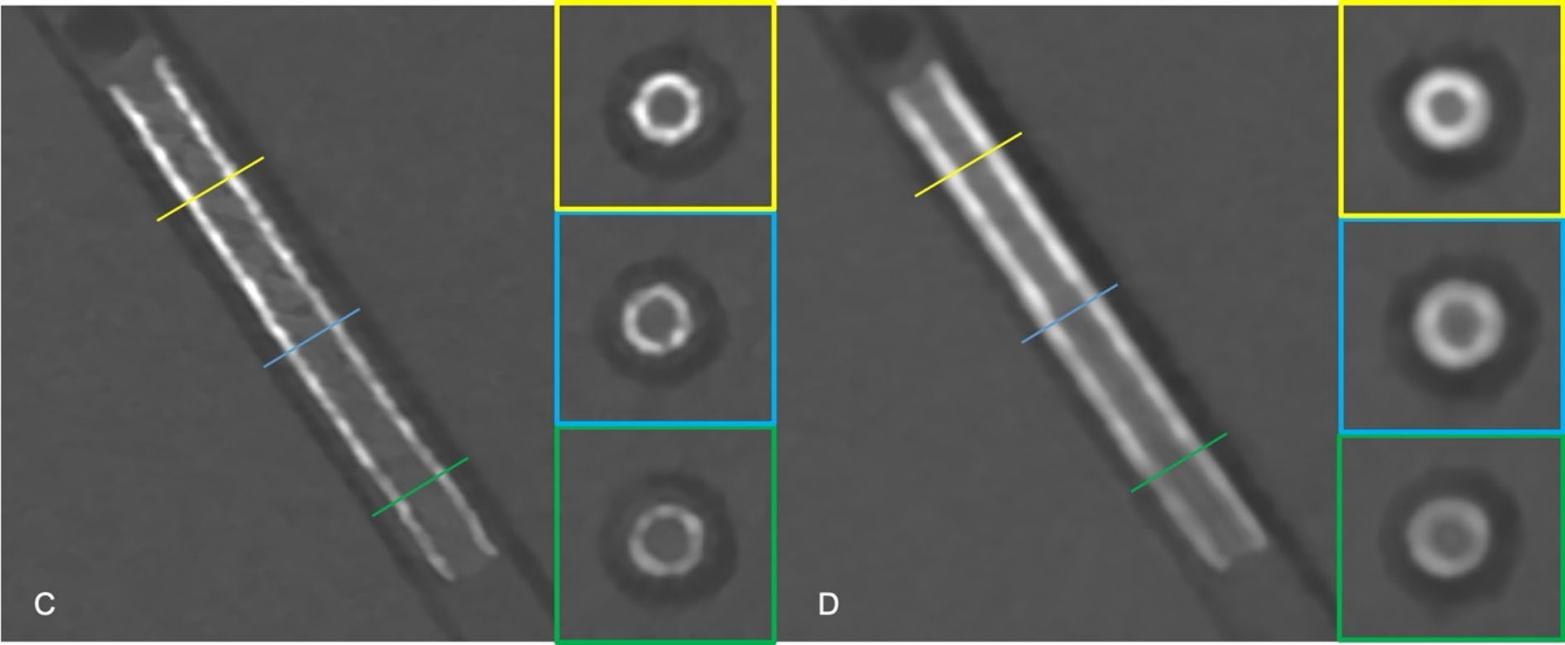
The image shows a 2.5 mm stent of the “Multilayer Series” in (**C**) Bv72 kernel reconstruction with a 0.2 mm slice thickness (“ultra- high resolution”) and (**D**) Bv56 kernel reconstruction with a 0.4 mm slice thickness. The yellow, blue, and green line correlate with the horizontal images on the right, showing the single- layer stent zone (green), the two- layer zone (blue) and the three- layer zone (yellow), respectively.)

All CT series were reconstructed from image raw data using a dedicated research software (ReconCT 16.0, Siemens Healthineers, Erlangen, Germany) with a 512 × 512 matrix size. For SR scan mode a virtual monoenergetic level of 70 keV, a quantum iterative reconstruction (QIR) strength of 4 and slice thickness/ increment of 0.4 mm were used. For scans in UHR mode, the same reconstruction settings without using spectral information were applied with a difference in slice thickness and increment, which was reduced to 0.2 mm. Based on the acquired data, three reconstruction series for each scanning mode (UHR and SR) were generated using specialized vascular kernels of progressively increasing sharpness (Bv56, and Bv72). See Figs. 3 and 4 for exemplary reconstructions with different kernels.

### Image analysis

All image series were transferred to a dedicated workstation for further analyses of quantitative and qualitative image parameters. All measurements were conducted using SyngoVia software (Syngo.via VB60A, Siemens Healthineers, Erlangen, Germany). The images were aligned in both, axial and sagittal orientations, allowing readers to scroll through the entire stent area. For both, the quantitative and qualitative assessment, two independent observers (D.O.B) and (E.H.) with cumulative 17 years of experience in cardiovascular CT imaging performed a blinded consensus read.

**Fig. 5:**
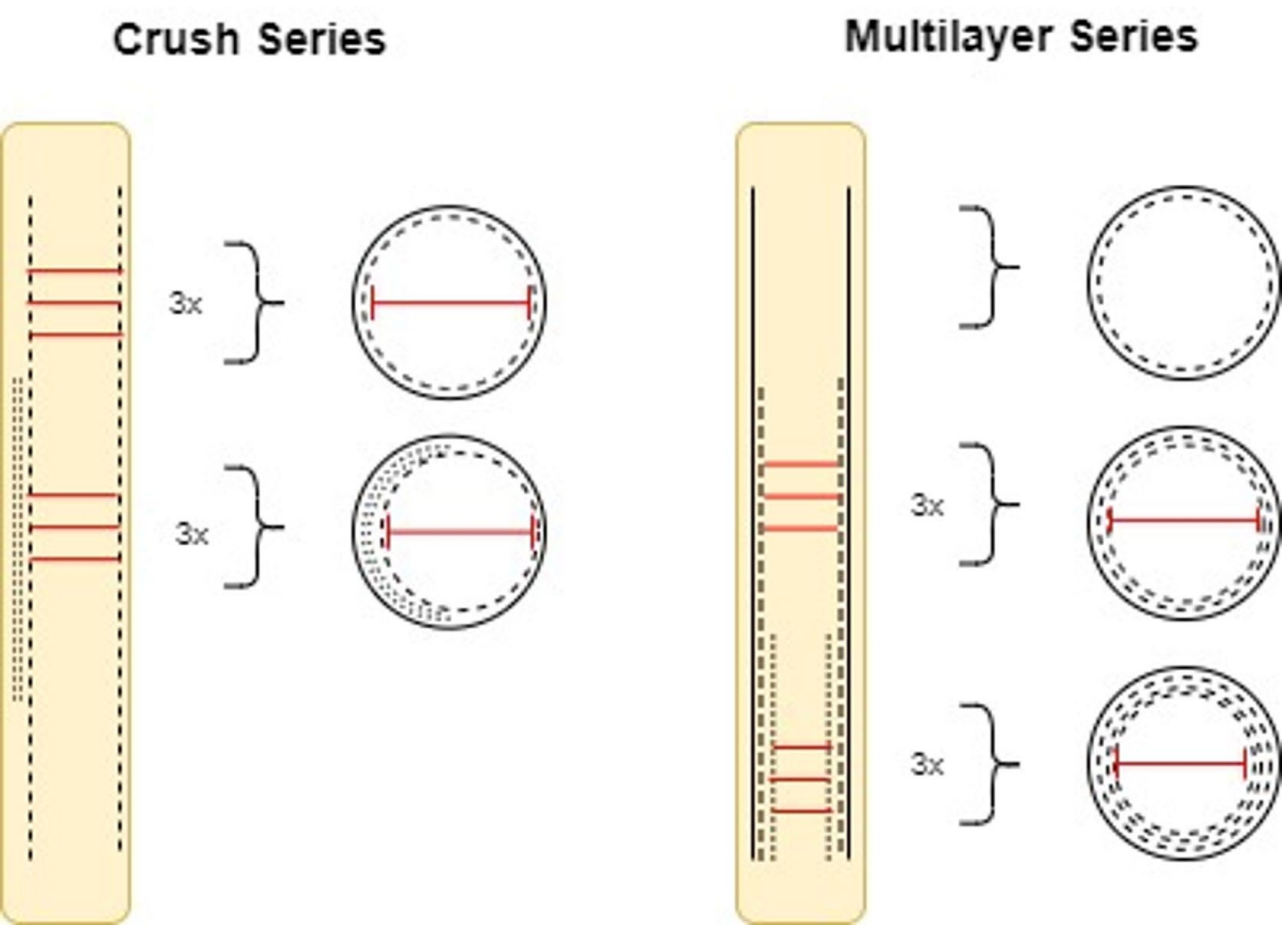
Assessment of In- Lumen visibility.

For quantitative image analysis, we measured the visible lumen diameter in three representative axial slices for each stent segment. These segments included the single- layer vs. crush- layer region in the crush series and the two-, and three- layer region in the multilayer series, respectively Figure 5. The quantitative or objective image analyses included the visible stent diameter, the attenuation in and outside the stent (both in absolute and relative numbers), as well as the signal to noise ratio.

Stent lumen diameter was measured following the method described by Gassenmaier et al.^14^, including the dark area adjacent to the stent struts. For every single measurement, we then calculated the percentage of visible lumen relative to the stent diameter by dividing the visible lumen diameter by the stent diameter.

Lumen attenuation was assessed in the same three representative axial slices using a region of interest (ROI) as large as possible but avoiding stent strut artifacts. Additionally, we measured the attenuation in three slices outside the stent but within the plastic tube filled with diluted contrast agent, to serve as a reference value. We then calculated the “delta stent attenuation” by subtracting the average outside stent attenuation from the inside stent attenuation. The delta stent attenuation is considered the stent related attenuation, i.e., the additional attenuation caused by the stent struts itself. Attenuation values were expressed as average Hounsfield Units (HU) and standard deviation within the ROI. Additionally, the change of attenuation inside the stent, as compared to outside the stent, was also calculated in percent.

To determine the presence of a predominant signal, defined as a signal- to- noise ratio (SNR) greater than 1:1, the SNR was calculated by dividing the attenuation within the stent by the standard deviation.

For qualitative image evaluation, multiple blinded readings were performed for each stent segment. Using a five- point Likert scale, as previously described^15^, five criteria were assessed subjectively: overall image quality, sharpness, perceived image noise, blooming, and diagnostic confidence. Image quality was graded as 1 = excellent (absence of artifacts), 2 = good (mild artefacts), 3 = fair (moderate artifacts), 4 = poor (pronounced artifacts), 5 = non- diagnostic (severe artifacts).

### Statistical analysis

Initial analyses focused on the single-layer zone in the Crush series, comparing all four kernel combinations (UHR Bv72 vs. SR Bv72, UHR Bv72 vs. SR Bv56, UHR Bv56 vs. SR Bv72, UHR Bv56 vs. SR Bv56; see Supplemental Table). Based on these findings, subsequent analyses in the Crush Zone and Multilayer 2/3 zones were restricted to a comparison against the most favorable kernel identified in the initial analysis (Tables 1, 2 for objective parameters, Tables 3,4 for subjective parameters). In the small stent subgroup, only the two top-performing kernels were compared (Tables 5, 6). As the single-layer configuration was already evaluated in the Crush series, the multilayer analysis included only the 2- and 3-layer zones.

Subjective image quality was evaluated using a 5-point Likert scale. As these qualitative data are ordinal and not normally distributed, all pairwise comparisons were analyzed using the non-parametric Mann–Whitney U test.

Normally distributed quantitative continuous variables are reported as mean ± standard deviation, while those with non- normal distribution are expressed as median and interquartile ranges. The Shapiro- Wilk test was employed to assess the normality of data distribution. Comparisons of quantitative variables across different reconstructions and kernels were conducted using the Mann- Whitney U test or t- test, as appropriate. To control for type I error inflation due to multiple testing, Bonferroni correction was applied. An adjusted p- value of ≤ 0.05 was considered indicative of statistically significant differences.

## Results

Quantitative and subjective results are summarized in Tables 1, 2 (objective), 3,4 (subjective), and 5, 6 (small-stent subgroup), with detailed kernel comparisons for single-layer and crushed configurations provided in Supplemental Table 1. Unless otherwise stated, p-values refer to two-sided tests as indicated in the tables.

### Quantitative image analysis

#### In- stent lumen visibility

Quantitative analysis demonstrated a statistically significant improvement of in- stent lumen visibility for the sharp Bv72 kernel reconstruction of the novel UHR- PCD mode in all regions of the stent, i.e., single- layer and crush zone. UHR (Bv72) increased in‑stent lumen visibility compared with SR across the Crush series [Supplemental Table 1]., with the effect most pronounced in small stents (≤ 3.0 mm)Table 5. In the small‑stent subgroup, lumen visibility increased in the single‑layer zone from 65.45 ± 1.91% (SR) to 77.11 ± 2.55% (UHR) and in the crushed zone from 60.67 ± 2.67% (SR) to 74.00 ± 2.67% (UHR), both *p* < 0.01 Table 5. Results for the full cohort show the same direction of effect Tables 1, 2.

### Stent attenuation changes

In the Crush series, our findings showed a positive correlation between the number of stent layers and stent attenuation, i.e., as the number of stent layers increased, a corresponding rise in stent attenuation values was observed. Stent attenuation changes were consistently kernel- dependent, with the Bv72 kernel yielding the lowest stent attenuation values.

Across configurations, the percentage change in stent attenuation increased with complexity (single‑layer < crushed < two‑layer < three‑layer), reflecting stronger blooming with more material Tables 1, 2; Supplemental Table 1. Kernel‑dependent patterns were observed. In the comparison of UHR (Bv72) vs. SR (Bv72), UHR produced lower attenuation changes in the single‑layer and crushed configurations, but higher values in two‑ and three‑layer settings. In contrast, when UHR (Bv72) was compared with SR (Bv56), UHR yielded lower or comparable attenuation changes across configurations Tables 1, 2; Supplemental Table 1.

### Signal- to- noise ratio (SNR)

Analysis of the signal- to- noise ratio (SNR) across the Crush series demonstrated a reconstruction kernel- independent higher SNR in SR scan mode compared to the novel UHR scan mode, reaching statistical significance in most comparisons. SNR was generally higher for SR than for UHR across most comparisons [Tables 1, 2]. Representative examples include the single‑layer zone: 3.11 ± 1.36 (UHR Bv72) vs. 5.04 ± 0.73 (SR Bv72), and the crushed zone: 3.28 ± 1.45 (UHR Bv72) vs. 5.82 ± 0.65 (SR Bv72) (both *p* < 0.01). This aligns with the expected noise penalty at 0.2‑mm slice thickness Tables 1, 2; Supplemental Table 1].

**Table 1:** Objective image quality metrics (Bv72 UHR vs. Bv72 SR).

Metric	SR (Bv72) mean ± SD	UHR (Bv72) mean ± SD	*p*-value
In stent lumen visibility (% of diameter) (1 Layer)	68.08 ± 4.30	79.03 ± 3.79	< 0.01
In stent lumen visibility (% of diameter) (Crush Zone)	63.62 ± 4.97	74.73 ± 2.51	< 0.01
In stent lumen visibility (% of diameter) (2 Layer)	64.56 ± 3.94	69.04 ± 4.22	0,11
In stent lumen visibility (% of diameter) (3 Layer)	63.48 ± 3.02	66.77 ± 4.26	0,08
Change in stent attenuation (%) (1 Layer)	88.89 ± 8.47	72.44 ± 15.28	0,01
Change in stent attenuation (%) (Crush Zone)	143.81 ± 16.88	95.82 ± 14.27	< 0.01
Change in stent attenuation (%) (2 Layer)	155.04 ± 28.29	195.64 ± 56.06	0,07
Change in stent attenuation (%) (3 Layer)	242.94 ± 86.90	265.24 ± 75.17	0,38
Signal to noise ratio (SNR) (1 Layer)	5.04 ± 0.73	3.11 ± 1.36	< 0.01
Signal to noise ratio (SNR) (Crush Zone)	5.82 ± 0.65	3.28 ± 1.45	< 0.01
Signal to noise ratio (SNR) (2 Layer)	5.05 ± 2.41	5.28 ± 1.95	0,82
Signal to noise ratio (SNR) (3 Layer)	5.05 ± 1.90	5.23 ± 1.26	0,81

All stent diameters (2.5, 3.0, 3.5 mm) were pooled. Measurements were performed at four configurations: single-layer (Crush series), crushed zone (Crush series), two-layer and three-layer (Multilayer series). Values are reported as mean ± SD; p-values from two-sided tests as applicable. Comparison in Table 1: UHR (Bv72) vs. SR (Bv72). UHR = ultra-high resolution photon-counting CT (0.2 mm slices); SR = standard-resolution photon-counting CT; BvXX = reconstruction kernel. Percentage change in stent attenuation is computed relative to the outside-stent reference (averaged from three measurements per section).

**Table 2:** Objective image quality metrics (Bv72 UHR vs. Bv56 SR).

Metric	SR (Bv56) mean ± SD	UHR (Bv72) mean ± SD	*p*-value
In stent lumen visibility (% of diameter) (1 Layer)	63.63 ± 4.47	79.03 ± 3.79	< 0.01
In stent lumen visibility (% of diameter) (Crush Zone)	62.18 ± 3.55	74.73 ± 2.51	< 0.01
In stent lumen visibility (% of diameter) (2 Layer)	64.12 ± 3.72	69.04 ± 4.22	0.08
In stent lumen visibility (% of diameter) (3 Layer)	61.08 ± 6.56	66.77 ± 4.26	0.04
Change in stent attenuation (%) (1 Layer)	102.76 ± 20.73	72.44 ± 15.28	< 0.01
Change in stent attenuation (%) (Crush Zone)	142.66 ± 29.01	95.82 ± 14.27	< 0.01
Change in stent attenuation (%) (2 Layer)	195.19 ± 65.91	195.64 ± 56.06	0.99
Change in stent attenuation (%) (3 Layer)	309.99 ± 119.28	265.24 ± 75.17	0.19
Signal to noise ratio (SNR) (1 Layer)	5.43 ± 2.81	3.11 ± 1.36	0.04
Signal to noise ratio (SNR) (Crush Zone)	3.41 ± 1.88	3.28 ± 1.45	0.88
Signal to noise ratio (SNR) (2 Layer)	7.46 ± 4.61	5.28 ± 1.95	0.21
Signal to noise ratio (SNR) (3 Layer)	7.10 ± 6.03	5.23 ± 1.26	0.79

All stent diameters (2.5, 3.0, 3.5 mm) were pooled. Measurements were performed at four configurations: single-layer (Crush series), crushed zone (Crush series), two-layer and three-layer (Multilayer series). Values are reported as mean ± SD; p-values from two-sided tests as applicable. Comparison in Table 1: UHR (Bv72) vs. SR (Bv56). UHR = ultra-high resolution photon-counting CT (0.2 mm slices); SR = standard-resolution photon-counting CT; BvXX = reconstruction kernel. Percentage change in stent attenuation is computed relative to the outside-stent reference (averaged from three measurements per section).

### Subjective image quality

Across the Crush and Multilayer series, UHR (Bv72) showed improved subjective image quality compared with SR reconstructions, as measured using a 5-point Likert scale. In particular, sharpness and blooming ratings were significantly better for UHR in both, the UHR Bv72 vs. SR Bv72 and the UHR Bv72 vs. SR Bv56 comparisons, while diagnostic confidence improved in several configurations [Tables 3,4]. Noise ratings were comparable in the crushed zone, but were higher (indicating more noise) for UHR in the multilayer configuration.

**Table 3:** Subjective image quality (Bv72 UHR vs. Bv72 SR).

Metric	SR (Bv72) median [IQR]	UHR (Bv72) median [IQR]	*p*-value
Sharpness (Multilayer)	1 [1]	1 [0]	< 0.01
Sharpness (Crush)	1 [1]	1 [0]	0.04
Noise (Multilayer)	1 [0]	2 [1]	< 0.01
Noise (Crush)	1 [0]	1 [0]	0.36
Blooming (Multilayer)	1 [1]	1 [0]	0.04
Blooming (Crush)	1 [0.25]	1 [0]	0.08
Diagnostic confidence (Multilayer)	1 [0]	2 [1]	< 0.01
Diagnostic confidence (Crush)	1 [0]	1 [0]	1.00
Overall image quality (Multilayer)	1 [1]	1 [1]	0.51
Overall image quality (Crush)	1 [0]	1 [0]	0.58

All stent diameters (2.5, 3.0, 3.5 mm) were pooled. Measurements were performed at four configurations: single-layer (Crush series), crushed zone (Crush series), two-layer and three-layer (Multilayer series). Values are median [IQR]; two-sided p-values from the Mann–Whitney U test. Comparison in Table 3: UHR (Bv72) vs. SR (Bv72). Likert scale (1 = excellent, 2 = good, 3 = fair, 4 = poor, 5 = non-diagnostic).

**Table 4:** Subjective image quality (Bv72 UHR vs. Bv56 SR).

Metric	SR (Bv56) median [IQR]	UHR (Bv72) median [IQR]	*p*-value
Sharpness (Multilayer)	2 [0]	1 [0]	< 0.01
Sharpness (Crush)	2 [0]	1 [0]	< 0.01
Noise (Multilayer)	2 [0]	2 [1]	< 0.01
Noise (Crush)	1 [0]	1 [0]	0.58
Blooming (Multilayer)	2 [0]	1 [0]	< 0.01
Blooming (Crush)	2 [1]	1 [0]	< 0.01
Diagnostic confidence (Multilayer)	2 [0]	2 [1]	0.03
Diagnostic confidence (Crush)	1 [0]	1 [0]	1.00
Overall image quality (Multilayer)	2 [0]	1 [1]	< 0.01
Overall image ql̥uality (Crush)	1.50 [1]	1 [0]	0.03

All stent diameters (2.5, 3.0, 3.5 mm) were pooled. Measurements were performed at four configurations: single-layer (Crush series), crushed zone (Crush series), two-layer and three-layer (Multilayer series). Values are median [IQR]; two-sided p-values from the Mann–Whitney U test. Comparison in Table 4: UHR (Bv72) vs. SR (Bv56). Likert scale (1 = excellent, 2 = good, 3 = fair, 4 = poor, 5 = non-diagnostic).

**Fig. 6:**
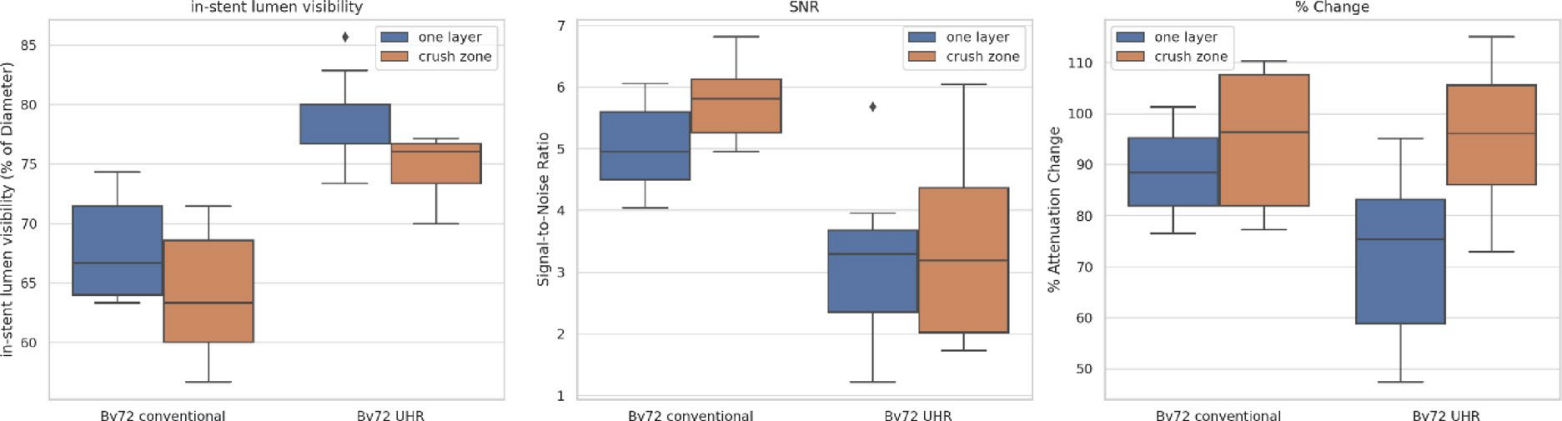
Quantitative image analysis for Bv72 image reconstruction comparing the novel UHR-PCD-CT with standard-resolution (SR) PCD-CT, displayed as “Bv72 conventional”. The three panels show the difference between one layer and crush zone parameters in-stent lumen visibility, signal-to-noise ratio (SNR) and % change of stent attenuation across standard and ultra-high-resolution PCD-CT.

The main subjective image quality results for the crushed series are summarized and presented as medians and quartiles in Fig. 6.

### Small stent subgroup

Findings in small stents mirrored the overall analysis: UHR (Bv72) improved lumen visibility and subjective image quality compared with SR, with kernel‑dependent behavior of attenuation change and the anticipated SNR trade‑off Tables 5, 6.

**Table 5:** Objective image quality metrics in small stents (Bv72 UHR vs. Bv72 SR).

Metric	SR (Bv72) mean ± SD	UHR (Bv72) mean ± SD	*p*-value
In stent lumen visibility (% of diameter) (1 Layer)	65.45 ± 1.91	77.11 ± 2.55	< 0.01
In stent lumen visibility (% of diameter) (Crush Zone)	60.67 ± 2.67	74.00 ± 2.67	< 0.01
In stent lumen visibilityy (% of diameter) (2 Layer)	62.55 ± 3.22	66.89 ± 3.29	0.04
In stent lumen visibility (% of diameter) (3 Layer)	61.89 ± 2.08	64.44 ± 3.09	0.25
Change in stent attenuation (%) (1 Layer)	85.08 ± 6.83	64.85 ± 12.20	< 0.01
Change in stent attenuation (%) (Crush Zone)	88.99 ± 11.36	96.15 ± 14.44	0.36
Change in stent attenuation (%) (2 Layer)	170.39 ± 20.30	229.41 ± 28.46	< 0.01
Change in stent attenuation (%) (3 Layer)	295.89 ± 44.25	314.45 ± 15.17	0.35
Signal to noise ratio (SNR) (1 Layer)	4.92 ± 0.78	2.66 ± 1.17	< 0.01
Signal to noise ratio (SNR) (Crush Zone)	5.62 ± 0.64	3.42 ± 1.79	0.02
Signal to noise ratio (SNR) (2 Layer)	6.39 ± 1.28	6.04 ± 1.35	0.48
Signal to noise ratio (SNR) (3 Layer)	4.81 ± 2.50	5.35 ± 2.06	0.69

Small stents (≤ 3.0 mm; 2.5 and 3.0 mm) were pooled. Measurements were performed at four configurations: single-layer (Crush series), crushed zone (Crush series), two-layer and three-layer (Multilayer series). Values are reported as mean ± SD; p-values from two-sided tests as applicable. Comparison in Table 1: UHR (Bv72) vs. SR (Bv72). UHR = ultra-high resolution photon-counting CT (0.2 mm slices); SR = standard-resolution photon-counting CT; BvXX = reconstruction kernel. Percentage change in stent attenuation is computed relative to the outside-stent reference (averaged from three measurements per section).

**Table 6:** Subjective image quality in small stents (Bv72 UHR vs. Bv72 SR).

Metric	SR (Bv72) median [IQR]	UHR (Bv72) median [IQR]	*p*-value
Sharpness (Multilayer)	1.50 [1]	1 [0]	< 0.01
Sharpness (Crush)	1.50 [1]	1 [0]	0.03
Noise (Multilayer)	1 [0]	2 [1]	< 0.01
Noise (Crush)	1 [0]	1 [0]	1.00
Blooming (Multilayer)	1 [0.25]	1 [0]	0.30
Blooming (Crush)	1 [1]	1 [0]	0.07
Diagnostic confidence (Multilayer)	1 [0]	1.50 [1]	< 0.01
Diagnostic confidence (Crush)	1 [0]	1 [0]	1.00
Overall image quality (Multilayer)	1 [1]	1.50 [1]	0.44
Overall image quality (Crush)	1 [0.25]	1 [0]	0.17

Small stents (≤ 3.0 mm; 2.5 and 3.0 mm) were pooled. Measurements were performed at four configurations: single-layer (Crush series), crushed zone (Crush series), two-layer and three-layer (Multilayer series). Values are median [IQR]; two-sided p-values from the Mann–Whitney U test. Comparison in Table 6: UHR (Bv72) vs. SR (Bv72). Likert scale (1 = excellent, 2 = good, 3 = fair, 4 = poor, 5 = non-diagnostic).

## Discussion

In this proof of concept ex- vivo study we compared two distinct CT scan modes (the novel UHR- PCD CT with ultra- thin 0.2 mm slice thickness and ultra- high resolution (UHR) and the standard resolution (SR)- PCD CT with 0.4 mm slice thickness) as well as sharp reconstruction kernels to evaluate image quality in complex stent situations. By simulating common bifurcation stenting strategies, including the *Culotte* and *Crush* techniques, we could show that the novel UHR- PCD CT using the Bv72 kernel significantly improved image sharpness, blooming, diagnostic confidence, and overall quality compared to the SR- PCD Bv56 kernel, while also outperforming the SR- PCD Bv72 reconstruction kernel in sharpness. Moreover, superior in- stent lumen visibility was demonstrated for the novel UHR- PCD Bv72 reconstruction across all multilayer- and crushed stent areas as compared to SR kernels, likely due to enhanced spatial resolution and reduced blooming artifacts.

However, the novel UHR-PCD Bv72 kernel yielded lower stent attenuation values and a lower signal-to-noise ratio (SNR) compared to SR scan modes, suggesting a trade-off between improved structural delineation and increased noise. Iterative reconstruction was applied to reduce image noise, and different reconstruction settings were evaluated to partially compensate for the lower SNR. Additional strategies—such as advanced model-based iterative reconstruction, post-reconstruction denoising filters, or deep learning–based algorithms—could be explored in future studies to further optimize the balance between noise reduction and preservation of fine image details.

A possible explanation for the lower SNR observed for some smooth kernel reconstructions in the crush stent zone, particularly in UHR mode, might be the asymmetrical distribution of stent layers in this region (e.g., multiple overlapping layers on one side and fewer layers on the other). This asymmetry can increase local signal heterogeneity and partial volume effects, partially offsetting the expected SNR benefit of smoother kernels. In contrast, the multilayer zone, with more symmetrical stacking of stent layers, shows the anticipated SNR improvement with smoother kernels.

Although recent guidelines do not yet recommend coronary computed tomography angiography (CCTA) as the preferred modality for evaluating symptomatic patients with prior coronary stenting [6; 16], a meta- analysis published in 2018 demonstrated that CCTA offers high accuracy for the assessment of most stents^17^. Several previously noted limitations—such as reduced accuracy in stents with diameters ≤ 3.0 mm, stent strut thickness ≥ 100 μm, or bifurcation stents resulting in multiple stent layers—have been significantly mitigated by advances in contemporary stent designs, novel reconstruction techniques (e.g., thin slices, sharp reconstruction kernels), and improvements in CT scanner technology^17–19^. As mentioned in the most recent *Society of Cardiovascular Computed Tomography* (SCCT) consensus statement, advances in spectral, high- definition and photon counting CT techniques were suspected to further improve the evaluation of intracoronary stents using CCTA^20^. However, to date no data exist evaluating complex stent scenarios using novel UHR- PCD CT.

To our knowledge, this is the first study utilizing UHR-PCD CT to evaluate multilayer configurations of small drug-eluting stents, demonstrating excellent diagnostic image quality. These results are consistent with findings by Decker et al. and Stein et al., who also reported significant improvements in the visualization of small stents (e.g., 2.5 mm), including better in-stent lumen visibility and sharpness, when using UHR acquisition mode and sharp reconstruction kernels on PCD-CT^10,21^. This study demonstrates that PCD- CT can reliably visualize up to three stent layers. Our findings emphasize that advanced image reconstruction techniques, i.e., by using advanced reconstruction kernels, play a key role in enhancing stent visualization by reducing blooming artifacts and improving edge definition.

For qualitative image analysis both, standard resolution (SR) and ultra- high- resolution (UHR) modes, provide high- quality, diagnostic images. The Bv72 kernel was found to offer notable advantages for delineating stent layers in both SR and UHR scan modes, particularly by increasing sharpness, reducing image blooming artifacts and increasing overall image quality. While some previous studies excluded sharper reconstruction kernels beyond Bv60 for stent assessment due to concerns over substantial increases in image noise and reductions in image quality^15^, our findings did not confirm these limitations. Although UHR scanning offered a modest additional improvement in sharpness, the SR mode—with its optimized reconstruction algorithm—already delivered excellent overall image quality and diagnostic confidence.

Notably, the UHR- Bv 72 kernel offered significant advantages over SR- Bv 56, with improved sharpness, reduced blooming, and enhanced overall image quality, reaffirming the value of high- resolution imaging paired with optimized kernel selection. Interestingly, when directly comparing UHR Bv 72 to SR- Bv 72, the improvements were limited to sharpness, with no significant differences observed in other parameters such as blooming, diagnostic confidence, or overall image quality. This lack of significance likely reflects the already excellent performance of the SR- Bv 72 kernel, which approached a ceiling effect in terms of image optimization for stent visualization.

This finding underscores the importance of kernel selection in maximizing the potential of imaging systems, especially in scenarios where UHR capabilities may not be readily available.

Furthermore, these results challenge the previous view that CT was inadequate for imaging complex stent configurations and confirm that modern PCD- CT systems are highly effective for detailed cardiac assessments.

Quantitative analysis of in- stent lumen visibility further highlights the advantages of ultra- high- resolution (UHR) imaging in stent evaluation, particularly when using the optimized Bv 72 reconstruction kernel. Enhanced in- stent lumen visibility with photon- counting detector CT (PCD- CT) in single stent layers has been previously demonstrated in several studies^22–25^.

However, to the best of our knowledge, a systematic evaluation of this technology in multiple stent layers, such as those encountered in bifurcation stenting, has not yet been performed, particularly in very small stents with diameters as low as 2.5 mm. In the crushed stent series, in- stent lumen visibility was significantly enhanced with ultra- high- resolution (UHR) imaging compared to SR- resolution mode, with statistically significant gains observed in both single- layer and multilayer (crushed) stent regions. UHR imaging provided superior delineation of the lumen within the stented segments, evident in both single- layer and multilayer (crushed) stent regions. This enhancement highlights UHR imaging’s superior ability to resolve fine details within the complex stent structures.

For the single- layer stent regions, UHR imaging achieved a lumen visibility of 79.0 ± 3.8% compared to 68.1 ± 4.3% with SR, reflecting its capacity to minimize artifacts and improve the accuracy of stent diameter measurements. Similar improvements were observed in the more challenging multilayer (crushed) stent regions, where UHR achieved 74.7 ± 2.5% visibility compared to 63.6 ± 5.0% with SR imaging. These findings indicate that the higher spatial resolution and enhanced signal processing of UHR imaging effectively mitigate the blooming and overlapping artifacts that often obscure lumen boundaries in stented regions, especially in areas with complex, multilayered configurations.

In the Crush series we observed a positive association between configuration complexity and attenuation change: the percentage change in stent attenuation increased from single-layer to crushed, two-layer, and three-layer setups (Tables 1, 2 small-stent subgroup in Table 5). Our attenuation metric is reported as % change relative to the outside-stent reference.

Kernel-dependent patterns were evident. For Bv72 UHR vs. Bv72 SR, UHR showed lower attenuation changes in the single-layer and crushed configurations, but higher values in the two- and three-layer settings. In contrast, for Bv72 UHR vs. Bv56 SR, UHR yielded lower or comparable attenuation changes across configurations.

Possible explanations include the higher spatial resolution of UHR (0.2-mm slices) and sharper reconstruction revealing partial-volume and overlap effects in multilayer regions more explicitly, whereas SR may blur these variations; differences in noise behavior between kernels that can modulate measured attenuation variability in complex geometries; and model-related factors (e.g., subtle asymmetries or misalignments in multilayer placement) that may disproportionately affect UHR measurements. These are hypotheses and should be interpreted with caution. Importantly, a smaller % change in attenuation should not be viewed as a disadvantage; rather, it indicates a more stable attenuation profile that supports improved in-stent lumen delineation—consistent with the objective and subjective image-quality results summarized in Tables 1, 2, 5.

This quantitative superiority complements the qualitative benefits observed, reinforcing UHR imaging as a preferred modality for coronary stent evaluation, particularly when paired with the Bv 72 reconstruction kernel.

The significantly improved in- stent lumen visibility observed with UHR imaging compared to SR mode is clinically meaningful, as it offers greater clarity in assessing lumen patency and stent integrity without the artifacts that often complicate SR imaging techniques^12^.

In the case of bifurcation stents or crushed stent configurations, where anatomical complexity increases the risk of incomplete stent expansion or malapposition, enhanced lumen visibility ensures more reliable evaluation of these critical areas. Detailed and accurate coronary visualization becomes even more important in cases in whom complex stent techniques are paired with high calcified plaque burden. In such cases, only non- invasive imaging with high lumen visibility – as it is the case for UHR- PCD CT – might be able to reduce the need for invasive coronary angiography, a procedure associated with procedural risks, patient discomfort, and higher costs. By providing clearer and more diagnostic images, UHR imaging may position CT as a more viable tool for routine follow- up and clinical decision- making, potentially transforming the paradigm of coronary stent assessment.

**Fig. 7:**
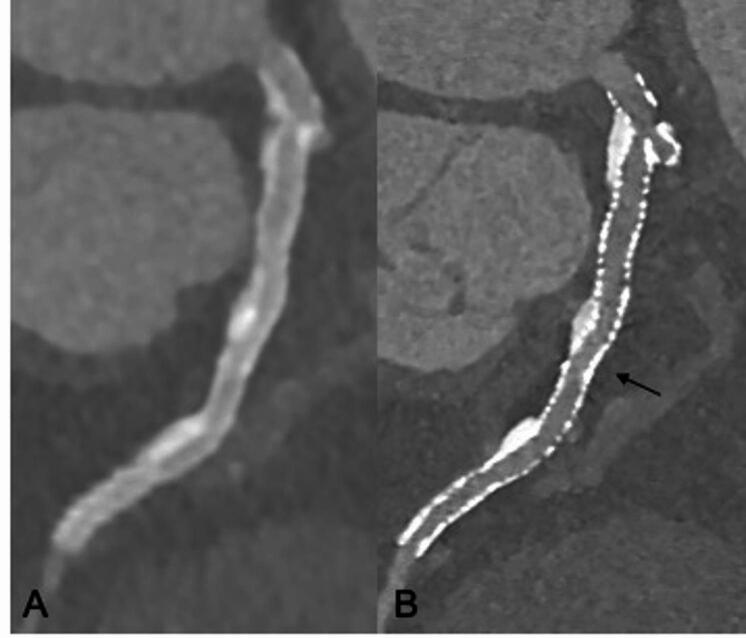
Stent Imaging on a novel Photon Counting Detector CT using (**A**) standard resolution scan mode and Bv 44 kernel vs. (**B**) Ultra High Resolution (UHR) scan mode and Bv 72 kernel. Due to the high spatial resolution and the sharp reconstruction kernels the stent structure is clearly visible – even in areas with multiple stent layers and the left main bifurcation – resulting in high diagnostic accuracy for stent assessment after a complex procedure.

The consistent improvement in both single- layer and multilayer regions emphasizes the robustness of UHR imaging for comprehensive stent assessment, offering a clear advantage in clinical scenarios where precise lumen evaluation is critical Figure 7 potentially reducing the need for invasive coronary angiography and thus reducing costs and potential procedure- related complications^26^.

### Limitations

Radiation dose was not assessed in this study, as all experiments were performed ex vivo using a phantom. While the phantom setup included tissue-equivalent attenuation, dose measurements were not feasible and reporting them could be misleading; therefore, potential dose implications of UHR acquisition should be interpreted with caution in clinical settings.

## Supplementary Information

Below is the link to the electronic supplementary material.

Supplementary Material 1

## Data Availability

The datasets used and/or analysed during the current study available from the corresponding author on reasonable request.

## Abbreviations

CAD: Coronary artery disease
CCTA: Coronary computed tomography angiography
CT: Computed tomography
EID-CT: Energy- integrating detector computed tomography
HU: Hounsfield units
IQR: Interquartile ranges
PCD- CT: Photon counting detector computed tomography
PCI: Percutaneous coronary intervention
UHR: Ultra high resolution
