# Supplementary material for: Multilayer and crushed stent visualization using photon- counting detector computed tomography – a proof of concept
Source: Sci Rep. 2025 Oct 27;15:37357. doi: 10.1038/s41598-025-25119-9 (PMC12559316; doi:10.1038/s41598-025-25119-9)
Supplement: Supplementary file 1 — Supplementary Material 1 [file 41598_2025_25119_MOESM1_ESM.docx]

|  |  |  |  |
| --- | --- | --- | --- |
| **Metric** | **SR**  **mean ± SD** | **UHR**  **mean ± SD** | **p-value** |
| **UHR (Bv56) vs SR (Bv56)** |  |  |  |
| In Stent Lumen Visibility (% of diameter) (1 Layer) | 63.63 ± 4.47 | 66.22 ± 5.27 | 0.24 |
| In Stent Lumen Visibility (% of diameter) (Crush Zone) | 62.18 ± 3.55 | 64.76 ± 6.60 | 0.86 |
| Change in Stent Attenuation (%) (1 Layer) | 102.76 ± 20.73 | 107.26 ± 24.70 | 0.54 |
| Change in Stent Attenuation (%) (Crush Zone) | 142.66 ± 29.01 | 143.81 ± 16.88 | 0.92 |
| Signal to Noise Ratio (SNR) (1 Layer) | 5.43 ± 2.81 | 5.56 ± 7.99 | 0.25 |
| Signal to Noise Ratio (SNR) (Crush Zone) | 3.41 ± 1.88 | 2.92 ± 0.69 | 0.48 |
| **UHR (Bv72) vs SR (Bv72)** |  |  |  |
| In Stent Lumen Visibility (% of diameter) (1 Layer) | 68.08 ± 4.30 | 79.03 ± 3.79 | <0.01 |
| In Stent Lumen Visibility (% of diameter) (Crush Zone) | 63.62 ± 4.97 | 74.73 ± 2.51 | <0.01 |
| Change in Stent Attenuation (%) (1 Layer) | 88.89 ± 8.47 | 72.44 ± 15.28 | 0.01 |
| Change in Stent Attenuation (%) (Crush Zone) | 143.81 ± 16.88 | 95.82 ± 14.27 | <0.01 |
| Signal to Noise Ratio (SNR) (1 Layer) | 5.04 ± 0.73 | 3.11 ± 1.36 | <0.01 |
| Signal to Noise Ratio (SNR) (Crush Zone) | 5.82 ± 0.65 | 3.28 ± 1.45 | <0.01 |
| **UHR (Bv56) vs SR (Bv72)** |  |  |  |
| In Stent Lumen Visibility (% of diameter) (1 Layer) | 68.08 ± 4.30 | 66.22 ± 5.27 | 0.53 |
| In Stent Lumen Visibility (% of diameter) (Crush Zone) | 63.62 ± 4.97 | 64.76 ± 6.60 | 0.82 |
| Change in Stent Attenuation (%) (1 Layer) | 88.89 ± 8.47 | 107.26 ± 24.70 | 0.05 |
| Change in Stent Attenuation (%) (Crush Zone) | 95.62 ± 13.45 | 143.81 ± 16.88 | <0.01 |
| Signal to Noise Ratio (SNR) (1 Layer) | 5.04 ± 0.73 | 5.56 ± 7.99 | <0.01 |
| Signal to Noise Ratio (SNR) (Crush Zone) | 5.82 ± 0.65 | 2.92 ± 0.69 | <0.01 |
| **UHR (Bv72) vs SR (Bv56)** |  |  |  |
| In Stent Lumen Visibility (% of diameter) (1 Layer) | 63.63 ± 4.47 | 79.03 ± 3.79 | <0.01 |
| In Stent Lumen Visibility (% of diameter) (Crush Zone) | 62.18 ± 3.55 | 74.73 ± 2.51 | <0.01 |
| Change in Stent Attenuation (%) (1 Layer) | 102.76 ± 20.73 | 72.44 ± 15.28 | <0.01 |
| Change in Stent Attenuation (%) (Crush Zone) | 142.66 ± 29.01 | 95.82 ± 14.27 | <0.01 |
| Signal to Noise Ratio (SNR) (1 Layer) | 5.43 ± 2.81 | 3.11 ± 1.36 | 0.04 |
| Signal to Noise Ratio (SNR) (Crush Zone) | 3.41 ± 1.88 | 3.28 ± 1.45 | 0.88 |
|  |  |  |  |

Supplemental Table 1: All stent diameters (2.5, 3.0, 3.5 mm) were pooled. Crush series only: measurements at two configurations — single-layer and crushed zone. Four kernel comparisons are shown within one table: UHR (Bv56) vs SR (Bv56), UHR (Bv72) vs SR (Bv72), UHR (Bv56) vs SR (Bv72), and UHR (Bv72) vs SR (Bv56). Values are mean ± SD with two-sided p-values as applicable. Percentage change in stent attenuation is computed relative to the outside-stent reference (three measurements per section). UHR = ultra-high-resolution photon-counting CT (0.2 mm slices); SR = standard-resolution photon-counting CT; BvXX = reconstruction kernel.

**Supplemental Table 1. Objective image quality metrics (kernel comparison Crush series)**
